# Predictive models for the occurrence of expansive intracranial hematomas and outcomes after surgical evacuation in patients with traumatic brain injury in Uganda: a prospective cohort study

**DOI:** 10.3389/fsurg.2026.1781033

**Published:** 2026-09-15

**Authors:** Larrey Kasereka Kamabu, Godfrey S. Bbosa, Ronald Oboth, Ssenyondwa John Baptist, Martin N. Kaddumukasa, Daniel Deng, Hervé Monka Lekuya, Louange Maha Kataka, Joel Kiryabwire, Moses Galukande, Martha Sajatovic, Mark Kaddumukasa, Anthony T. Fuller, Michael M. Haglund

**Affiliations:** 1 Department of Surgery, Neurosurgery, College of Medicine, Makerere University, Kampala, Uganda; 2 Faculty of Medicine, Université Catholique du Graben, Butembo, Democratic Republic of the Congo; 3 Neurological Surgery, New Deal Sarl Hospital, International Clinic for Advanced Medicine in Kivu (CIMAK), Goma, Democratic Republic of the Congo; 4 Faculty of Medicine, Université Catholique de Louvain, Brussels, Belgium; 5 Department of Pharmacology & Therapeutics, Makerere University College of Health Sciences, Kampala, Uganda; 6 Department of Medicine, School of Medicine, College of Health Sciences, Makerere University, Kampala, Uganda; 7 Duke Global Neurosurgery and Neurology, Duke University, Durham, NC, United States; 8 Directorate of Surgical Services, Neurosurgical Unit, Mulago National Referral Hospital, Kampala, Uganda; 9 Neurological and Behavioral Outcomes Center, University Hospitals Cleveland Medical Center & Case Western Reserve University School of Medicine, Cleveland, OH, United States

**Keywords:** expansive intracranial hematoma, hematoma expansion, machine learning, neurosurgical outcomes, predictive modeling, traumatic brain injury, Uganda

## Abstract

**Background:**

Hematoma expansion is a frequent and clinically important complication of traumatic intracranial hemorrhage and is associated with poor functional outcomes. Early identification of patients at risk of expansive intracranial hematomas (EIH) remains challenging, particularly in low-resource settings.

**Objective:**

To determine the prevalence of EIH and develop exploratory predictive models for EIH occurrence and neurosurgical outcomes among patients with traumatic brain injury (TBI) in Uganda.

**Methods:**

We conducted a prospective cohort study among adult patients with TBI and radiologically confirmed intracranial hematomas at Mulago National Referral Hospital. EIH was defined using serial non-contrast computed tomography as hematoma growth greater than 33% or an absolute increase greater than 6 mL within 72 h of injury. Firth bias-reduced logistic regression was used for sparse binary outcomes. EIH prediction was evaluated using patient-level fitted probabilities, five-fold cross-validation, bootstrap optimism correction, calibration, and the Brier score; LASSO, elastic-net logistic regression, and random forest were compared as exploratory prediction approaches. Statistical significance was set at *p* < 0.05.

**Results:**

Among 324 patients, EIH occurred in 192 patients, representing 59.3% of the cohort. Patients with EIH had significantly poorer quality of life at 3 and 6 months postoperatively (*p* < 0.010). For EIH prediction, the Firth bias-reduced model showed an apparent AUC of 0.854 and a bootstrap optimism-corrected AUC of 0.845. In five-fold cross-validation, pooled out-of-fold AUCs were 0.835 for LASSO, 0.837 for elastic net, and 0.804 for random forest, indicating no predictive advantage of random forest over penalized logistic regression. Key predictors included subdural hematoma, diffuse axonal injury, systolic and diastolic blood pressure, and the interaction between skull fracture and subdural hematoma. Models for postoperative outcomes were exploratory and internally validated only.

**Conclusion:**

EIH was highly prevalent among patients with TBI in Uganda and was associated with poorer postoperative recovery. A parsimonious Firth model based on routinely available clinical and radiological variables showed strong internally validated discrimination, while penalized logistic models performed at least as well as random forest in cross-validation. These prediction models remain exploratory and require external multicenter validation before clinical implementation.

## Introduction

Traumatic brain injury (TBI) is a major global public health problem and a leading cause of death and long-term neurological disability, particularly in low- and middle-income countries (1). Worldwide, TBI affects an estimated 69 million individuals annually and is responsible for approximately 5 million deaths each year (2). Notably, more than 93% of TBI-related deaths occur in low- and middle-income countries (LMICs), where health systems face significant resource constraints (1). Road traffic crashes are a major global cause of traumatic injury and an important cause of TBI, particularly among young adults in low- and middle-income countries (3). In Uganda, mortality among patients with TBI is reported to be 4.2 times higher than that observed in the United States, underscoring profound disparities in trauma care and outcomes (4–6). Beyond mortality, TBI represents a major cause of long-term neurological sequelae, particularly among young and economically productive populations, thereby contributing substantially to social and economic burden (7). In Uganda, the burden of TBI is substantial, largely driven by road traffic accidents, motorcycle-related injuries, delayed presentation, and limited access to specialized neurosurgical care.

TBI encompasses a broad spectrum of primary and secondary brain injuries, including skull fractures, cerebral contusions, diffuse axonal injury, subdural hematoma, epidural hematoma, intracerebral hemorrhage, and subarachnoid hemorrhage (8). Among these, expansive intracranial hematoma (EIH) represents one of the most clinically important and time-sensitive complications (9). EIH is commonly defined as an increase in hematoma volume greater than 33% or an absolute increase greater than 6 mL on follow-up neuroimaging (9, 10). Hematoma expansion may worsen mass effect, increase intracranial pressure, accelerate neurological deterioration, and contribute to poor functional recovery (9).

Early identification of patients at high risk of EIH is therefore essential (9). In resource-limited settings, this challenge is even greater because repeat neuroimaging, neurosurgical monitoring, intensive care capacity, and timely operative intervention may be constrained (11). Conventional clinical decision-making often relies on neurological examination, admission Glasgow Coma Scale score, blood pressure, hematoma type, skull fracture pattern, and surgeon judgment (9–11). However, these variables may interact in complex and nonlinear ways that are not always captured by traditional risk stratification approaches.

Several prognostic models have been developed for TBI and intracranial hemorrhage, but many were derived from high-income settings and may not be directly applicable to LMIC trauma systems (12). In addition, existing models often focus on mortality or global functional outcome rather than hematoma expansion and surgical evacuation outcomes (9). There remains a need for context-specific predictive tools that use readily available clinical and radiological variables to support early identification of high-risk patients to EIH.

Machine learning approaches have gained increasing interest in clinical prediction because they can evaluate multidimensional data, identify nonlinear patterns, and explore interactions between predictors (13–15). These methods may be particularly useful for complex conditions such as TBI, where outcomes are influenced by combinations of clinical severity, radiological findings, physiological parameters, timing of care, and operative factors (16). However, machine learning models derived from single-center datasets require cautious interpretation, transparent reporting, internal, and external validation before they can be used for routine clinical decision-making (17, 18).

To date, limited evidence exists on predictive modeling for EIH and neurosurgical evacuation outcomes among patients with TBI in Uganda. Therefore, this study aimed to determine the prevalence of EIH, identify clinical and radiological predictors, and develop internally validated exploratory predictive models for EIH occurrence and postoperative neurosurgical outcomes among adult patients with TBI managed at Mulago National Referral Hospital (MNRH).

## Material and methods

### Study design

We conducted a prospective cohort study between June 2021 and June 2023 to evaluate the occurrence of expansive intracranial hematoma (EIH), identify clinical and radiological predictors, and develop exploratory predictive models for EIH occurrence and neurosurgical evacuation outcomes among adults with traumatic brain injury (TBI).

### Study setting

The study was conducted at Mulago National Referral Hospital (MNRH) in Kampala, Uganda. MNRH is the largest public tertiary hospital in Uganda and serves as a major national referral center for trauma and neurosurgical emergencies (19). The Accident and Emergency Department manages an average of 64 urgent neurosurgical evacuations for intracranial hemorrhage related to acute TBI each month (20).

Eligible patients were recruited from the Accident and Emergency Department and followed through operative theatres, neurosurgical wards, and outpatient neurosurgery clinics.

### Study population

The study population included adult patients aged 18 years or older with TBI and radiologically confirmed intracranial hematoma on non-contrast brain computed tomography (CT). Patients were recruited after initial emergency resuscitation and neurosurgical assessment.

### Participant selection criteria

Patients were eligible if they met the following criteria:

- Age ≥18 years
- Post-resuscitation Glasgow Coma Scale score between 4 and 14
- Radiologically confirmed traumatic intracranial hematoma
- Enrollment within 24 h of hospital presentation
- Eligibility for cranial surgical intervention
- Availability of baseline and follow-up CT imaging for assessment of hematoma evolution

Patients were excluded if they had known thrombocytopenia, documented coagulation disorders, anticoagulant use, pregnancy, or inability to obtain informed consent from the patient or next of kin before surgical intervention.

### Neuroimaging assessment and definition of EIH

All enrolled patients underwent baseline non-contrast brain CT after initial emergency resuscitation and clinical stabilization. Baseline CT imaging was obtained at presentation or within 24 h of hospital admission. Follow-up CT imaging was routinely performed within 72 h after injury to assess hematoma evolution. Earlier repeat CT imaging was obtained when there was neurological deterioration, worsening Glasgow Coma Scale score, new focal neurological deficit, pupillary change, or clinical concern for raised intracranial pressure.

EIH was defined as an increase in traumatic intracranial hematoma volume greater than 33% or an absolute hematoma growth greater than 6 mL on follow-up CT compared with the baseline scan (20).

Hematoma volume was estimated on axial CT images using standard radiological measurements. For hematomas with a relatively regular ellipsoid shape, the ABC/2 method was used, where A represented the greatest hemorrhage diameter, B the diameter perpendicular to A, and C the approximate craniocaudal depth estimated from the number and thickness of CT slices containing hemorrhage. For irregular, multilobar, or multicompartment hemorrhages, measurements were reviewed jointly and interpreted in relation to serial CT findings and neurosurgical assessment.

Baseline and follow-up CT scans were reviewed within the clinical care pathway by an experienced neurosurgeon and a consultant radiologist. For irregular, multilobar, or multicompartment hematomas, discrepant interpretations were resolved by consensus. A random subset of 65 paired baseline/follow-up CT examinations was independently reread by a second experienced reader without access to the original EIH classification. Agreement for EIH classification was 89.2%, with Cohen's *κ* = 0.777. Reliability for follow-up hematoma-volume measurement was excellent (ICC([2,1]) = 0.994).

### Study procedures and variables

Participants were followed longitudinally from admission through surgery, inpatient postoperative care, and outpatient follow-up for up to 6 months. Trained research assistants collected data using Research Electronic Data Capture (REDCap).

Collected variables included demographic characteristics, clinical parameters, laboratory findings, radiological features, surgical characteristics, and postoperative outcomes.

Demographic variables included age, sex, occupation, marital status, residence type, and geographic region. Clinical variables included Glasgow Coma Scale score, blood pressure parameters, neurological status, and timing of presentation. Laboratory variables included baseline hematological and biochemical indices. Radiological variables included hematoma type, anatomical location, hematoma volume, skull fracture pattern, diffuse axonal injury, midline shift when available, and temporal evolution on serial CT imaging.

Surgical variables included type of cranial surgery, timing of surgical evacuation, and surgical site. Surgical timing was categorized as early if performed within 24 h after injury, late if performed between 25 and 72 h, and delayed if performed after 72 h. In regression models, the 25–72-hour (late) category served as the reference because it represented the intermediate and clinically typical timing stratum against which earlier and more delayed intervention could be compared.

### Outcomes

The primary outcome was occurrence of EIH on serial CT imaging.

Secondary outcomes included all-cause mortality, postoperative complications, quality of life, and functional outcome after neurosurgical evacuation. Postoperative complications included early post-traumatic seizures, coma, cerebral edema, wound infection, pneumonia, cerebrospinal fluid leakage, subdural hygroma, nutritional deficits, and other clinically documented complications.

Quality of life was assessed using the Quality of Life after Brain Injury questionnaire at follow-up, including 3 and 6 months postoperatively. Functional outcome was assessed using the Glasgow Outcome Scale during follow-up. Favorable functional outcome was defined as Glasgow Outcome Scale score >3, and unfavorable functional outcome as Glasgow Outcome Scale score ≤3.

### Statistical analysis

Continuous variables were summarized as mean ± standard deviation for normally distributed data and median with interquartile range for non-normally distributed data. Categorical variables were summarized as frequencies and percentages.

Missing-data handling. The submitted analysis output did not include a variable-level missingness table. Analyses were therefore based on available complete observations for variables entering each model. No values were imputed. Before final resubmission, the authors reported the exact proportion of missingness for each key predictor from the source dataset and confirmed the analytic denominator for every multivariable model.

At the bivariate level, associations between candidate predictors and EIH occurrence were assessed using the chi-square test or Fisher's exact test for categorical variables and Student's *t*-test or non-parametric tests for continuous variables, as appropriate.

Multivariable models were selected according to outcome type. Because sparse outcome categories and complete or quasi-complete separation were identified in postoperative models, binary outcomes were reanalyzed using Firth bias-reduced penalized logistic regression. Surgical-location categories were collapsed into clinically meaningful single-region vs. multiregion groups when required to avoid sparse cells. Adjusted ORs were reported with two-sided 95% CIs and *p* values. The mortality model was deliberately restricted because only 42 deaths occurred, thereby reducing model instability.

Candidate variables were chosen using clinical relevance and the prespecified study framework. For the EIH model, systolic and diastolic blood pressure, subdural hematoma, diffuse axonal injury, skull fracture, and the skull-fracture × subdural-hematoma interaction were retained. For postoperative outcomes, parsimonious Firth models were used and sparse anatomical categories were collapsed. Late surgery (25–72 h) served as the timing reference because it represented the intermediate and most clinically interpretable comparison category. The variables used in the final models were complete in the certified analysis dataset; no imputation was required.

### Machine learning model development

Machine-learning analyses were conducted as exploratory prediction modeling. The final EIH predictor set comprised six features: systolic blood pressure, diastolic blood pressure, subdural hematoma, diffuse axonal injury, skull fracture, and the skull-fracture × subdural-hematoma interaction. Random forest was compared with LASSO and elastic-net logistic regression using stratified five-fold cross-validation. Discrimination was summarized by AUC and probabilistic accuracy by the Brier score.

Internal validation included five-fold cross-validation and nonparametric bootstrap optimism correction. For the EIH Firth model, patient-level fitted probabilities from the final model were used to construct the receiver operating characteristic curve (Figure 2). The apparent AUC was 0.854 (bootstrap 95% CI 0.814–0.893) and the optimism-corrected AUC was 0.845. Calibration was evaluated graphically and by calibration intercept (0.004), calibration slope (1.038), and Brier score (0.153). For mortality, the apparent AUC was 0.860, mean bootstrap optimism was 0.026, optimism-corrected AUC was 0.834, and Brier score was 0.088.

Because no independent external cohort was available, all validation was internal. Five-fold pooled out-of-fold AUCs were 0.835 for LASSO, 0.837 for elastic net, and 0.804 for random forest. Penalized logistic regression therefore matched or exceeded random-forest discrimination, and the prediction results are interpreted as exploratory.

The predictive models developed in this study were intended for risk stratification and hypothesis generation rather than immediate clinical deployment. External validation in independent cohorts is required before routine clinical use.

## Results

### Study flow of participants

During the study period, 1,500 patients with traumatic brain injury were screened for eligibility (Figure 1). Of these, 324 patients (21.6%) fulfilled the inclusion criteria and were enrolled in the study. Among the enrolled patients with traumatic intracranial hematomas, 192 (59.3%) developed expansive intracranial hematoma (EIH), whereas 132 (40.7%) did not (Figure 1; Table 1).

**Figure 1:**
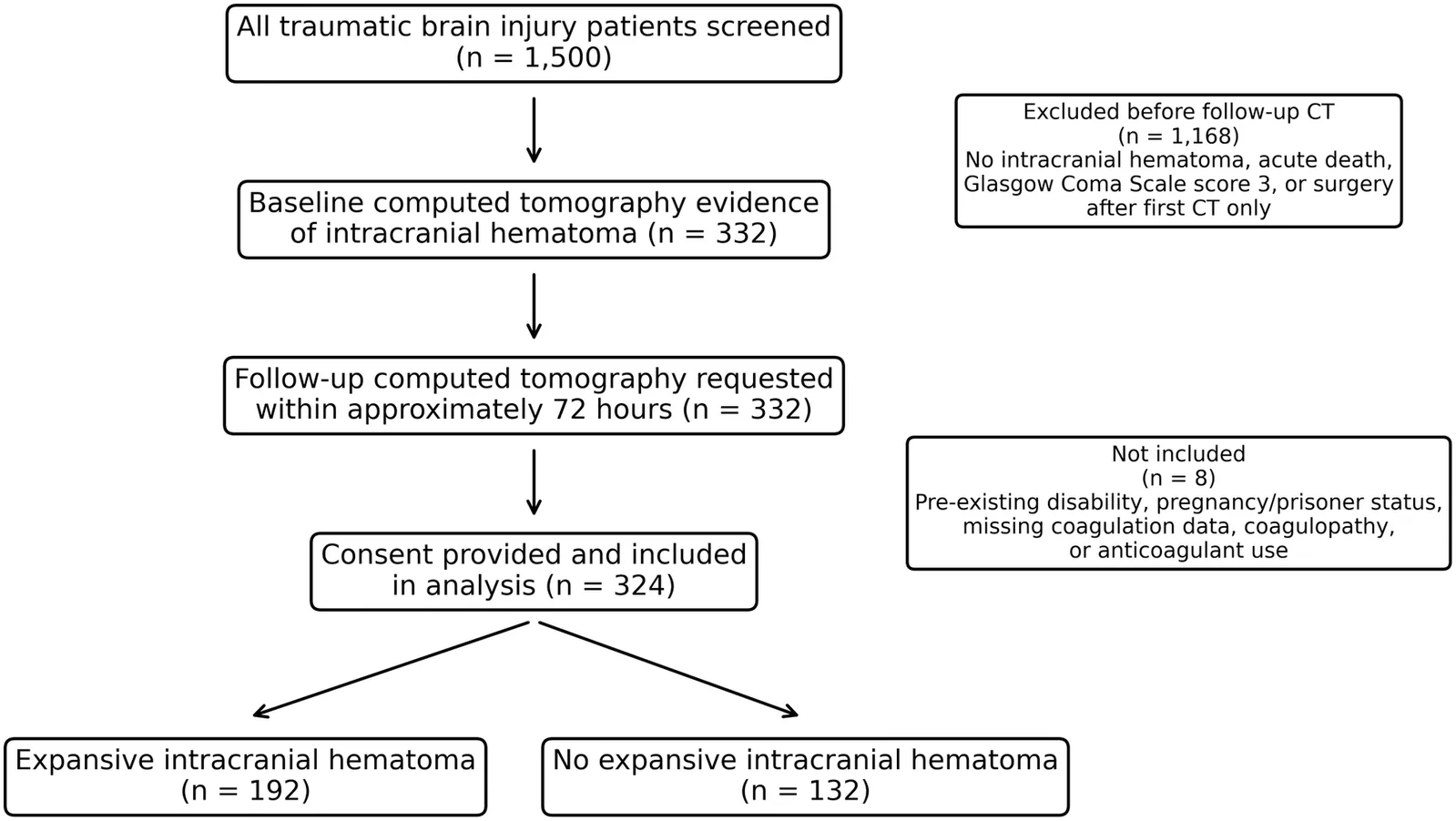
Flow chart of participant screening, eligibility assessment, enrollment, and expansive intracranial hematoma classification.

**Table 1:** Baseline demographic and clinical characteristics of patients with and without expansive intracranial hematoma.

Variables	No EIH, *n* (%)	EIH, *n* (%)	Total, *n* (%)	*p*-value
*n* (%)	132 (40.7)	192 (59.3)	324 (100.0)	
Age in complete years, mean (sd)	30.5 (14.0)	42.3 (17.9)	37.5 (17.4)	<0.001
Gender, *n* (%)				
Female	24 (18.2)	39 (20.3)	63 (19.4)	
Male	108 (81.8)	153 (79.7)	261 (80.6)	0.634
Patients' occupation, *n* (%)				
Farming	11 (8.3)	26 (13.5)	37 (11.4)	
Family business	16 (12.1)	38 (19.8)	54 (16.7)	
Government worker	6 (4.5)	8 (4.2)	14 (4.3)	
Industry worker	3 (2.3)	5 (2.6)	8 (2.5)	
Boda rider	72 (54.5)	80 (41.7)	152 (46.9)	
Taxi driver	23 (17.4)	29 (15.1)	52 (16.0)	
Other, specify	1 (0.8)	6 (3.1)	7 (2.2)	0.136
Residence type/location, *n* (%)				
Rural	78 (59.1)	106 (55.2)	184 (56.8)	
Urban	54 (40.9)	86 (44.8)	140 (43.2)	0.488
Region, *n* (%)				
Central	95 (72.0)	135 (70.3)	230 (71.0)	
Eastern	10 (7.6)	9 (4.7)	19 (5.9)	
Northern	8 (6.1)	27 (14.1)	35 (10.8)	
Western	19 (14.4)	21 (10.9)	40 (12.3)	0.088
Matrimonial state, *n* (%)				
Unmarried	62 (47.0)	65 (33.9)	127 (39.2)	
Married	70 (53.0)	127 (66.1)	197 (60.8)	0.017

### Baseline demographic and clinical characteristics

The baseline demographic and clinical characteristics were summarized in Table 1. The mean age of the study population was 37.5 ± 17.4 years. Patients who developed EIH were significantly older than those without EIH (42.3 ± 17.9 vs. 30.5 ± 14.0 years; *p* < 0.001). Overall, 261 patients (80.6%) were male and 63 (19.4%) were female. Motorcycle riders (“boda-boda”) represented the largest occupational group (152/324, 46.9%), while 184 patients (56.8%) resided in rural areas. Marital status differed significantly between the two groups (*p* = 0.017), whereas sex, occupation, residence, and geographic region were not significantly associated with EIH occurrence (all *p* > 0.05) (Table 1).

### Prevalence of expansive intracranial hematoma

Among the 324 patients included in the analysis, 192 developed EIH, corresponding to an overall prevalence of 59.3%, while 132 patients (40.7%) had no evidence of hematoma expansion on follow-up CT imaging (Table 1).

### Predictors of expansive intracranial hematoma

Firth bias-reduced logistic regression identified systolic blood pressure per 10-mmHg increase (OR 2.04, 95% CI 1.65–2.51, *p* < 0.001), diastolic blood pressure per 10-mmHg increase (OR 0.60, 95% CI 0.46–0.79, *p* < 0.001), subdural hematoma (OR 12.15, 95% CI 4.85–30.42, *p* < 0.001), diffuse axonal injury (OR 12.88, 95% CI 5.00–33.19, *p* < 0.001), and the skull-fracture × subdural-hematoma interaction (OR 0.22, 95% CI 0.07–0.70, *p* = 0.011) as independent predictors of EIH. Skull fracture alone was not significant (OR 1.45, 95% CI 0.69–3.05, *p* = 0.332) (Table 2).

**Table 2:** Firth bias-reduced logistic regression predictors of expansive intracranial hematoma.

Predictor	OR	95% CI	*p*-value
SBP per 10 mmHg	2.04	1.65–2.51	<0.001
DBP per 10 mmHg	0.60	0.46–0.79	<0.001
Subdural hematoma	12.15	4.85–30.42	<0.001
Diffuse axonal injury	12.88	5.00–33.19	<0.001
Skull fracture	1.45	0.69–3.05	0.332
Skull fracture × SDH	0.22	0.07–0.70	0.011

EIH, expansive intracranial hematoma; SDH, subdural hematoma; DAI, diffuse axonal injury; OR, odds ratio; CI, confidence interval.

### Machine learning model performance for EIH prediction

Penalized and machine-learning models were compared using five-fold cross-validation. Pooled out-of-fold AUC was 0.835 for LASSO, 0.837 for elastic net, and 0.804 for random forest. The final EIH Firth model generated the patient-level ROC curve shown in Figure 2, with an apparent AUC of 0.854 (bootstrap 95% CI 0.814–0.893) and a bootstrap optimism-corrected AUC of 0.845. Calibration showed a Brier score of 0.153, intercept 0.004, and slope 1.038 (Supplementary Figure S1).

**Figure 2:**
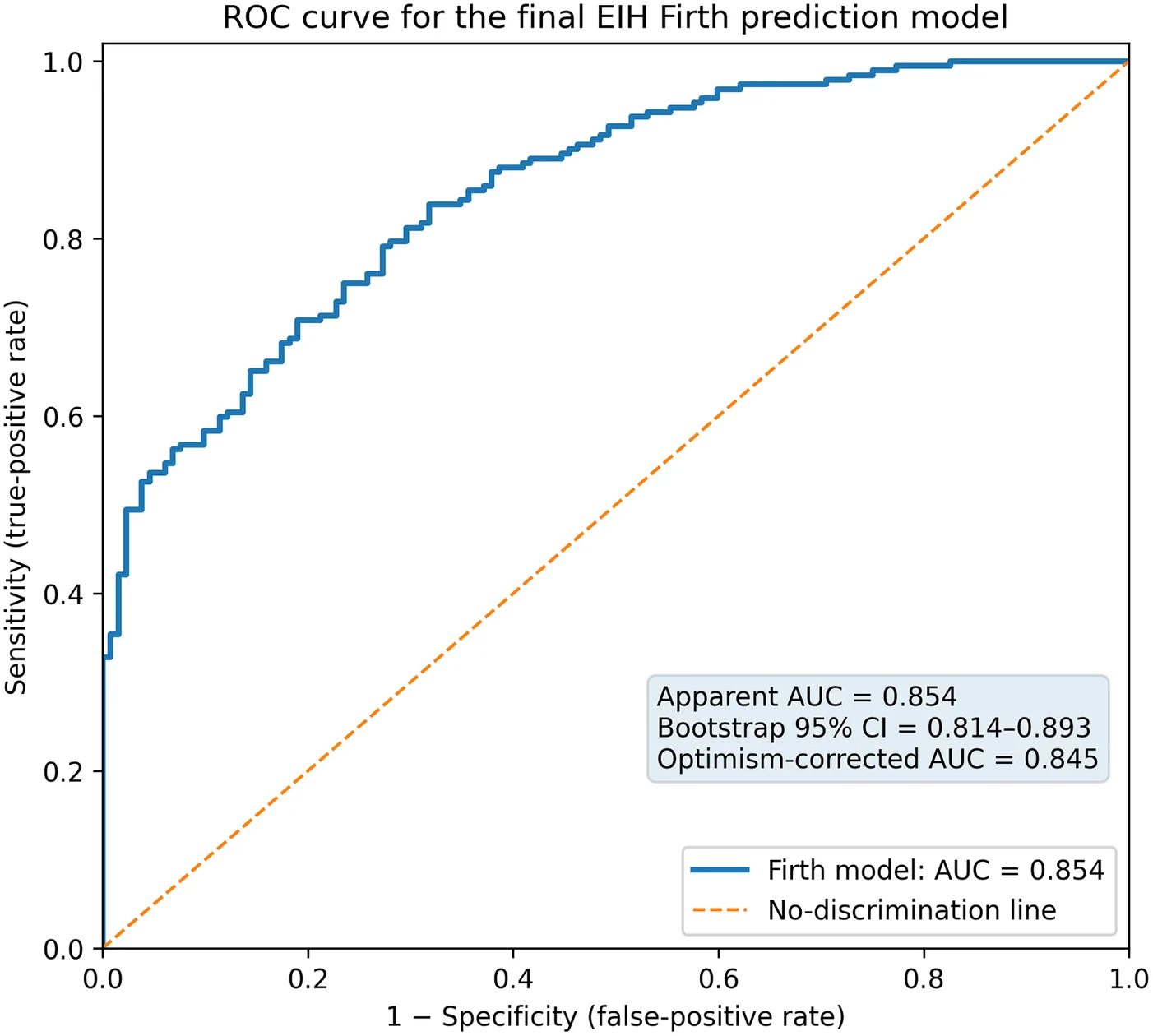
Receiver operating characteristic curve for the final firth model predicting expansive intracranial hematoma. The curve was generated directly from patient-level predicted probabilities from the final fitted model. The apparent AUC was 0.854 (bootstrap 95% CI 0.814–0.893); the bootstrap optimism-corrected AUC was 0.845.

### Postoperative complications and clinical outcomes

Postoperative complications and clinical outcomes are summarized in Table 3. Overall, 188 patients (58.0%) experienced at least one postoperative complication, whereas 136 (42.0%) had no documented complication. The most common complications were early post-traumatic seizures (51/324, 15.7%), followed by brain swelling (38/324, 11.7%), coma (38/324, 11.7%), infection (16/324, 4.9%), nutritional deficit (13/324, 4.0%), pneumonia (11/324, 3.4%), cerebrospinal fluid leakage (9/324, 2.8%), and subdural hygroma (4/324, 1.2%).

**Table 3:** Postoperative complications and clinical outcomes among patients with and without expansive intracranial hematoma.

Complications, *n* (%)	No EIH, *n* = 132 (40.7%)	EIH, *n* = 192 (59.3%)	Total, *n* = 324 (100.0%)	*p*-Value
No	53 (40.2)	83 (43.2)	136 (42.0)	
Yes	79 (59.8)	109 (56.8)	188 (58.0)	0.581
Infection (Y), *n* (%)	8 (6.1)	8 (4.2)	16 (4.9)	0.439
Pneumonia (infection of the lungs) (Y), *n* (%)	8 (6.1)	3 (1.6)	11 (3.4)	0.028
Early PTS (Y), *n* (%)	22 (16.7)	29 (15.1)	51 (15.7)	0.704
Brain swelling (Y), *n* (%)	11 (8.3)	27 (14.1)	38 (11.7)	0.115
Leakage of cerebrospinal fluid) (Y), *n* (%)	4 (3.0)	5 (2.6)	9 (2.8)	0.816
Subdural hygroma	3 (2.3)	1 (0.5)	4 (1.2)	0.161
Nutrition deficit	5 (3.8)	8 (4.2)	13 (4.0)	0.864
Coma (Y), *n* (%)	13 (9.8)	25 (13.0)	38 (11.7)	0.383
Death	20 (15.1)	22 (11.4)	42 (12.9)	
Alive	116 (87.9)	175 (91.1)	282 (88.4)	0.073

Overall, 42 patients (12.9%) died during follow-up, whereas 282 patients (87.1%) survived. Survival was observed in 116/132 (87.9%) patients without EIH and 175/192 (91.1%) patients with EIH, although this difference did not reach statistical significance (*p* = 0.073) (Table 3).

### Predictors of intraoperative complications

Factors associated with intraoperative complications are presented in Table 4 and Figure 3. In the Firth model, decompressive craniectomy (OR 2.68, 95% CI 1.24–5.80, *p* = 0.012), delayed surgery >72 h vs. late surgery (OR 3.62, 95% CI 1.73–7.57, *p* < 0.001), and three-region surgical involvement (OR 4.78, 95% CI 2.11–10.83, *p* < 0.001) were independently associated with higher odds of intraoperative complications.

**Table 4:** Firth bias-reduced logistic regression for intraoperative complications.

Predictor	OR	95% CI	*p*-value
Craniotomy	0.53	0.23–1.19	0.125
Decompressive craniectomy	2.68	1.24–5.80	0.012
Early surgery vs. late	1.37	0.74–2.53	0.318
Delayed surgery vs. late	3.62	1.73–7.57	<0.001
Two-region site	1.34	0.75–2.39	0.318
Three-region site	4.78	2.11–10.83	<0.001
EIH	1.42	0.81–2.49	0.218

**Figure 3:**
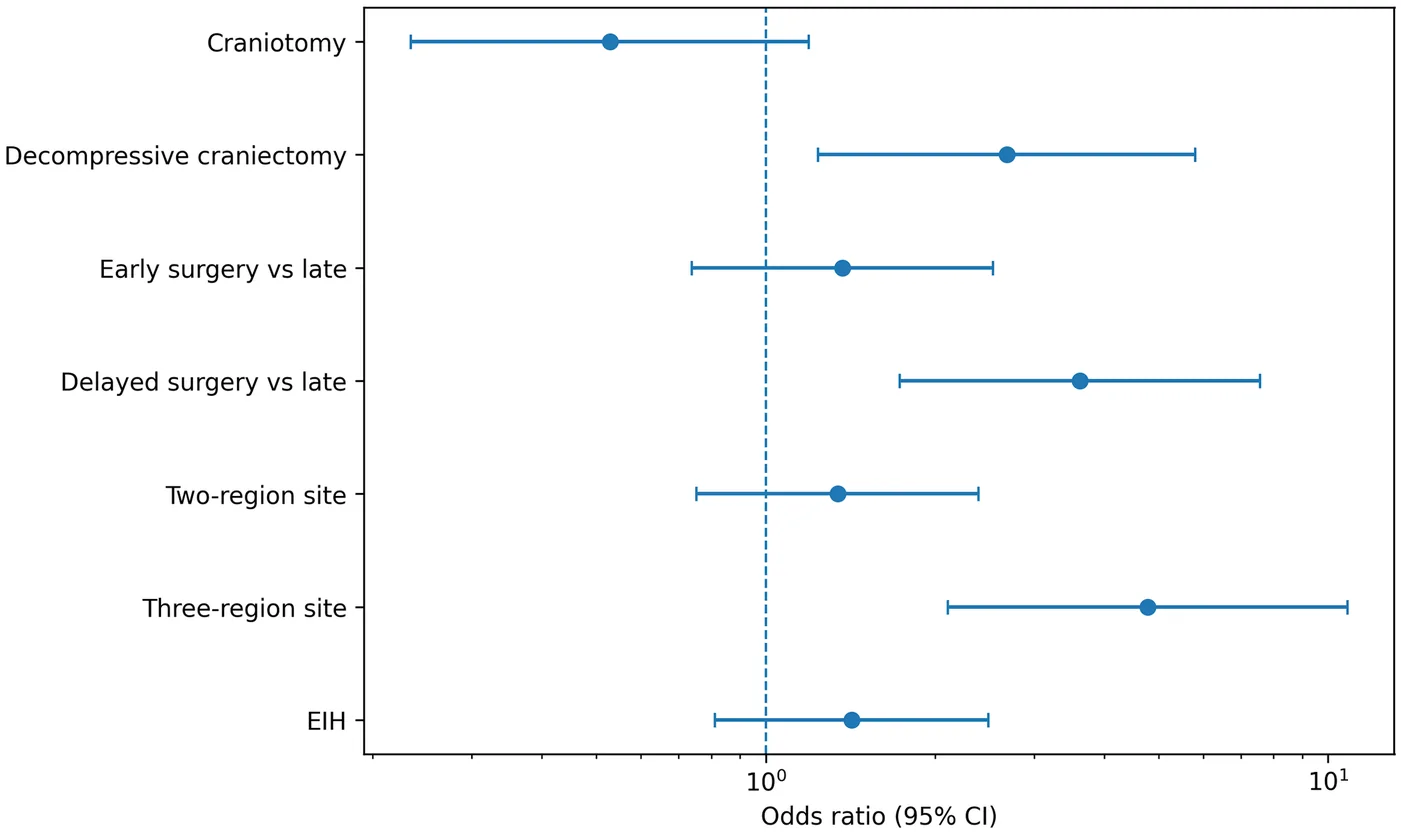
Firth bias-reduced regression for intraoperative complications. Points show adjusted ORs and horizontal bars 95% CIs.

### Predictors of mortality after neurosurgical evacuation

The parsimonious Firth mortality model is presented in Table 5 and Figure 4. Mortality was independently associated with age per 10-year increase (OR 1.40, 95% CI 1.12–1.76, *p* = 0.003), EIH (OR 4.24, 95% CI 1.57–11.41, *p* = 0.004), intraoperative complication (OR 2.58, 95% CI 1.11–6.00, *p* = 0.027), craniotomy (OR 8.14, 95% CI 1.48–44.63, *p* = 0.016), and delayed surgery >72 h (OR 2.74, 95% CI 1.08–6.93, *p* = 0.033). Apparent AUC was 0.860 and bootstrap optimism-corrected AUC was 0.834.

**Table 5:** Parsimonious firth bias-reduced logistic regression for mortality after neurosurgical evacuation.

Predictor	OR	95% CI	*p*-value
Age per 10 years	1.40	1.12–1.76	0.003
EIH	4.24	1.57–11.41	0.004
Intraoperative complication	2.58	1.11–6.00	0.027
Craniotomy	8.14	1.48–44.63	0.016
Decompressive craniectomy	1.22	0.20–7.49	0.831
Early surgery vs. late	1.04	0.43–2.52	0.931
Delayed surgery vs. late	2.74	1.08–6.93	0.033
Multiregion site	0.80	0.39–1.66	0.554

**Figure 4:**
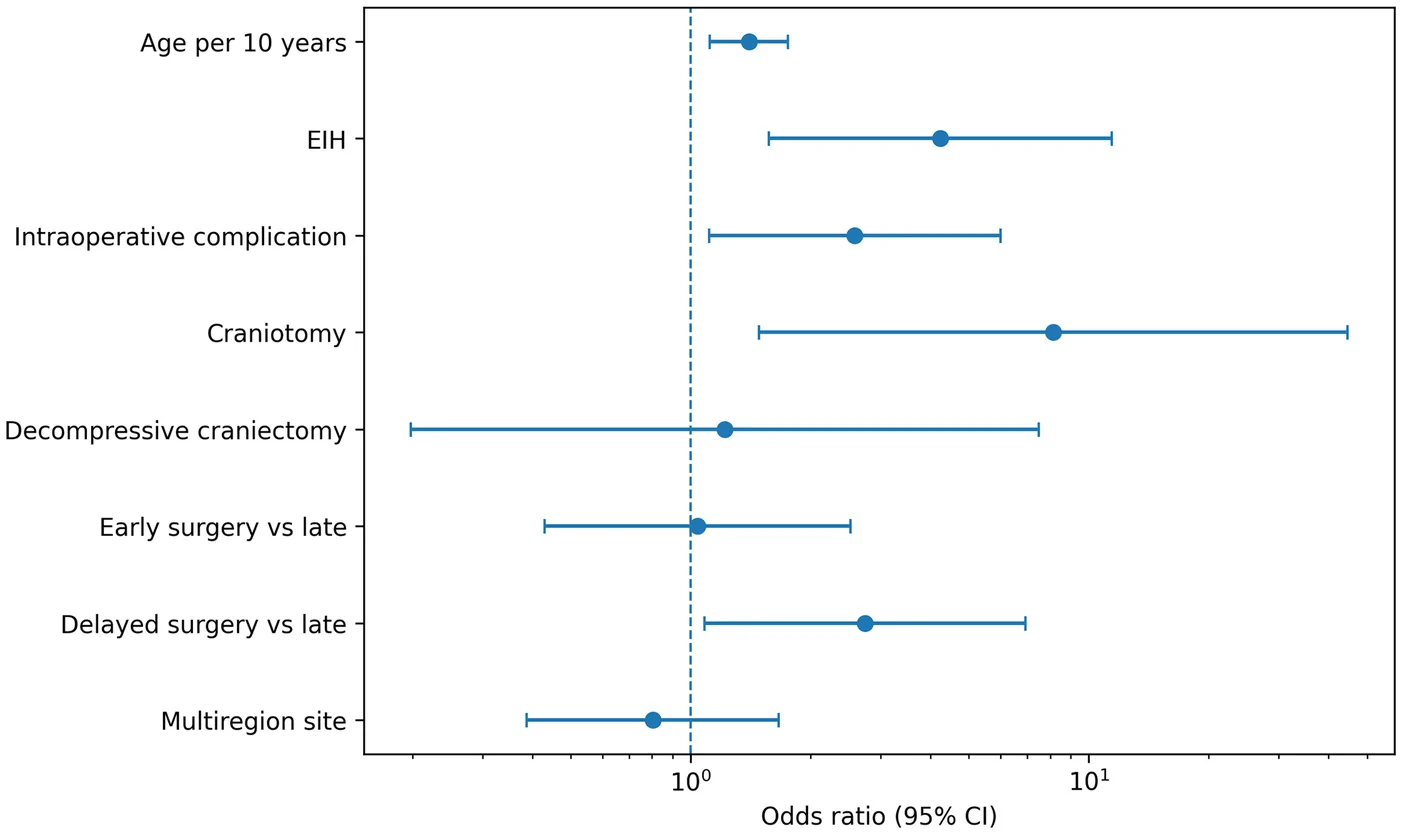
Parsimonious firth bias-reduced mortality model. Points show adjusted ORs and horizontal bars 95% CIs.

### Quality-of-life outcomes

Quality of life following neurosurgical evacuation is summarized in Table 6 and illustrated in Figure 5. QoLIBRI scores improved progressively during follow-up in both groups. However, patients without EIH consistently demonstrated higher scores than those with EIH. At 90 days, the mean QoLIBRI score was 79.4 ± 9.5 in the non-EIH group compared with 76.3 ± 10.4 in the EIH group (*p* = 0.011). At 6 months, the corresponding scores were 88.0 ± 8.6 and 85.0 ± 9.5, respectively (*p* = 0.010) (Table 6; Figure 5).

**Table 6:** Quality-of-life scores after neurosurgical evacuation among patients with and without expansive intracranial hematoma.

Quality of life after surgical evacuation in TBI patients	No EIH, *n* (%)	EIH, *n* (%)	Total, *n* (%)	*p*-value
*n* (%)	132 (40.7)	192 (59.3)	324 (100.0)	
Overall QoLIBRI first 24 h, mean (sd)	50.0 (15.3)	49.0 (16.8)	49.4 (16.2)	0.596
Overall QoLIBRI 30 days, mean (sd)	61.6 (19.6)	62.2 (17.2)	61.9 (18.2)	0.771
Overall QoLIBRI 90 days, mean (sd)	79.4 (9.5)	76.3 (10.4)	77.5 (10.1)	0.011
Overall QoLIBRI 6 months, mean (sd)	88.0 (8.6)	85.0 (9.5)	86.0 (9.3)	0.010

6 months corresponds to 180 days of follow-up. QoLIBRI, Quality of Life after Brain Injury.

**Figure 5:**
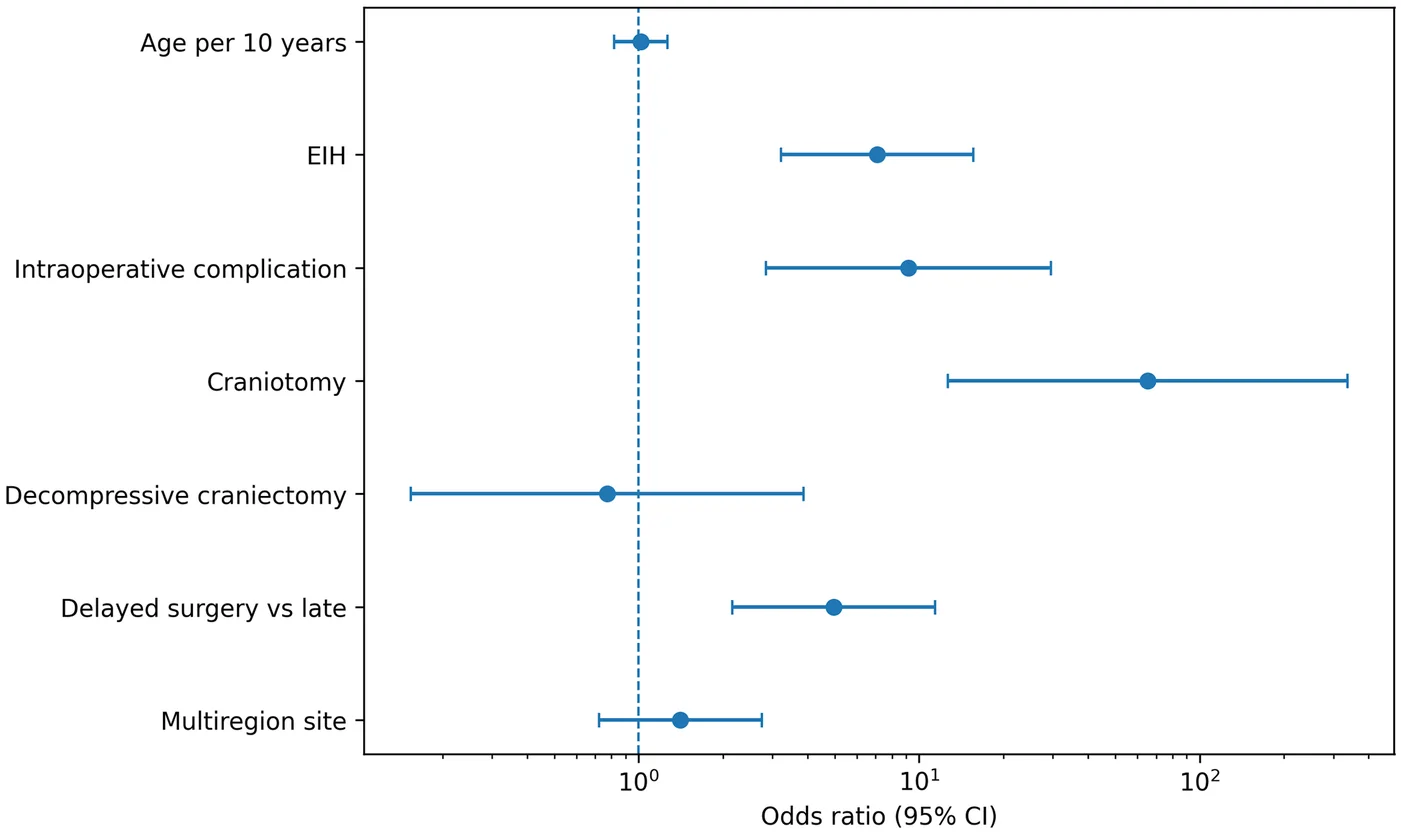
Firth bias-reduced regression for unfavorable quality of life at 6 months. Points show adjusted ORs and horizontal bars 95% CIs.

Firth multivariable analysis identified EIH (OR 7.08, 95% CI 3.21–15.60, *p* < 0.001), intraoperative complication (OR 9.14, 95% CI 2.84–29.46, *p* < 0.001), craniotomy (OR 65.31, 95% CI 12.67–336.53, *p* < 0.001), and delayed surgery >72 h (OR 4.96, 95% CI 2.16–11.39, *p* < 0.001) as independent predictors of unfavorable quality of life at 6 months (Table 7; Figure 5).

**Table 7:** Firth bias-reduced logistic regression for unfavorable quality of life at 6 months.

Predictor	OR	95% CI	*p*-value
Age per 10 years	1.02	0.82–1.27	0.880
EIH	7.08	3.21–15.60	<0.001
Intraoperative complication	9.14	2.84–29.46	<0.001
Craniotomy	65.31	12.67–336.53	<0.001
Decompressive craniectomy	0.77	0.15–3.87	0.754
Delayed surgery vs. late	4.96	2.16–11.39	<0.001
Multiregion site	1.41	0.72–2.75	0.315

Outcome harmonized as unfavorable quality of life at 6 months after neurosurgical evacuation. The large craniotomy OR should be interpreted cautiously and likely reflects confounding by indication and residual injury severity rather than a causal adverse effect of craniotomy.

### Functional outcomes

Factors associated with unfavorable functional outcome are summarized in Table 8 and Figure 6. EIH (OR 4.04, 95% CI 2.23–7.31, *p* < 0.001), intraoperative complication (OR 3.55, 95% CI 1.91–6.60, *p* < 0.001), and craniotomy (OR 6.25, 95% CI 2.43–16.08, *p* < 0.001) were independently associated with unfavorable functional outcome. Decompressive craniectomy showed a borderline association (OR 2.52, 95% CI 0.96–6.62, *p* = 0.060).

**Table 8:** Firth bias-reduced logistic regression for unfavorable functional outcome.

Predictor	OR	95% CI	*p*-value
Age per 10 years	1.08	0.92–1.28	0.328
EIH	4.04	2.23–7.31	<0.001
Intraoperative complication	3.55	1.91–6.60	<0.001
Craniotomy	6.25	2.43–16.08	<0.001
Decompressive craniectomy	2.52	0.96–6.62	0.060
Delayed surgery vs. late	1.59	0.83–3.02	0.160
Multiregion site	1.16	0.69–1.96	0.564

**Figure 6:**
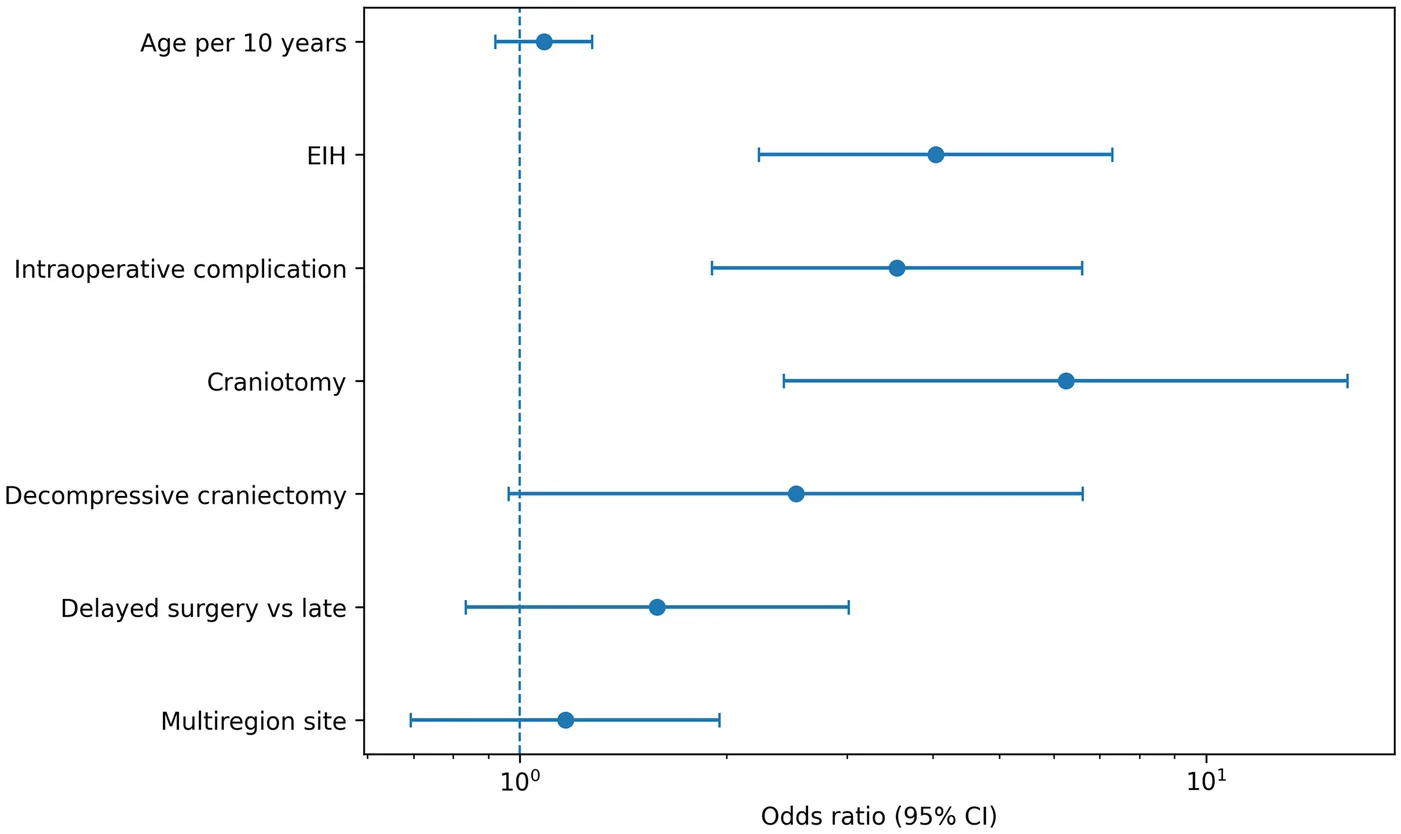
Firth bias-reduced regression for unfavorable functional outcome. Points show adjusted ORs and horizontal bars 95% CIs.

## Discussion

This prospective cohort study provides one of the largest contemporary evaluations of expansive intracranial hematoma (EIH) among patients undergoing neurosurgical management for traumatic brain injury (TBI) in sub-Saharan Africa. By integrating conventional multivariable regression with penalized and machine-learning techniques, this study identified clinically meaningful predictors of hematoma expansion, developed internally validated exploratory predictive models for EIH occurrence, and examined associations with postoperative outcomes. Several important findings emerged. First, EIH occurred in nearly three-fifths of patients with traumatic intracranial hematomas. Second, systolic blood pressure, diastolic blood pressure, subdural hematoma, diffuse axonal injury, and the interaction between skull fracture and subdural hematoma independently predicted hematoma expansion. Third, the final Firth model showed strong apparent discrimination (AUC = 0.854, bootstrap 95% CI 0.814–0.893) with limited bootstrap optimism (optimism-corrected AUC = 0.845). In five-fold cross-validation, elastic net (AUC = 0.837) and LASSO (AUC = 0.835) modestly outperformed random forest (AUC = 0.804), indicating that greater model complexity did not confer a predictive advantage in this cohort. Collectively, these findings support parsimonious, clinically interpretable models based on routinely available clinical and radiological variables as promising candidates for future external validation in resource-constrained settings.

The observed prevalence of EIH (59.3%) was considerably higher than that reported in many previous studies, where hematoma expansion rates have generally ranged from approximately 13% to 38%, depending on patient selection, imaging protocols, injury severity, and definitions of expansion (17, 21). Several factors may explain this difference. First, our study exclusively enrolled patients with traumatic intracranial hematomas requiring neurosurgical evaluation, representing a population with more severe injuries than those included in many mixed TBI cohorts. Second, serial CT imaging performed according to institutional neurosurgical protocols allowed systematic detection of hematoma progression within the critical early period after injury. Third, delayed presentation following road traffic injuries, limited prehospital care, and restricted access to advanced trauma systems in Uganda may contribute to ongoing secondary hemorrhage before definitive treatment (5, 19). These findings emphasize that the epidemiology of EIH differs substantially across healthcare systems and highlight the importance of generating locally derived evidence rather than relying exclusively on data from high-income countries. Such context-specific evidence is particularly valuable for informing neurotrauma management strategies in low-resource settings, where delayed diagnosis and treatment remain common.

Among the identified predictors, SDH emerged as the strongest independent radiological determinant of EIH, increasing the odds of hematoma expansion more than approximately twelvefold. This observation is consistent with previous investigations demonstrating that SDH reflects extensive tearing of bridging veins and severe primary traumatic injury, thereby predisposing patients to continued bleeding and progressive hematoma enlargement (17). Because SDH is frequently accompanied by cortical contusions, cerebral edema, and elevated intracranial pressure, it may also serve as a surrogate marker of overall injury severity rather than representing an isolated pathological process. Consequently, patients presenting with acute SDH should undergo close neurological observation and timely repeat CT imaging, particularly during the first 72 h after injury, when hematoma expansion is most likely to occur.

Diffuse axonal injury was another important independent predictor of EIH. Although DAI is traditionally regarded as a diffuse white matter injury resulting from rotational acceleration forces, it frequently coexists with multifocal vascular disruption and widespread microhemorrhages, reflecting severe biomechanical injury to the brain. Previous neuroimaging studies have demonstrated that patients with DAI often exhibit more extensive secondary injury, prolonged intracranial hypertension, and poorer neurological outcomes than patients without axonal injury (22, 23). Our findings therefore suggest that DAI should not only be viewed as a marker of neurological severity but also as an indicator of increased vulnerability to progressive hemorrhagic expansion. The incorporation of DAI into predictive models may therefore improve early risk stratification compared with reliance on hematoma characteristics alone.

Blood pressure parameters also contributed significantly to EIH prediction. Higher systolic blood pressure was independently associated with increased odds of hematoma expansion, whereas increasing diastolic blood pressure demonstrated an inverse association after multivariable adjustment. Elevated systolic blood pressure following TBI may reflect sympathetic hyperactivity, impaired cerebral autoregulation, or physiological compensation in response to elevated intracranial pressure. Excessive systolic pressure can promote continued bleeding from injured intracranial vessels and facilitate hematoma enlargement, particularly during the acute post-traumatic period. Similar associations between elevated systolic blood pressure and hematoma progression have been described in studies of both traumatic and spontaneous intracranial hemorrhage (24, 25). In contrast, the observed relationship with diastolic blood pressure is more difficult to interpret biologically and may reflect complex interactions between cerebral perfusion pressure, vascular compliance, injury severity, and treatment interventions. Because observational studies cannot establish causality, these findings should be interpreted cautiously and warrant further investigation in larger multicenter cohorts.

An interesting observation was the significant interaction between skull fracture and SDH. Although skull fracture alone was not independently associated with EIH after multivariable adjustment, its interaction with SDH significantly modified the risk of hematoma expansion. This finding suggests that combinations of anatomical injuries may provide greater prognostic information than individual variables considered separately. Such interactions are often difficult to identify using conventional statistical analyses but may become more apparent when interaction terms are systematically evaluated or when machine-learning algorithms capture nonlinear relationships among predictors. The clinical implication is that isolated radiological findings should not be interpreted independently; instead, integrated assessment of multiple injury characteristics may provide a more accurate estimate of an individual patient's risk profile.

One strength of this study was the direct comparison of conventional bias-reduced regression with penalized logistic and machine-learning approaches. Elastic net and LASSO matched or exceeded random-forest discrimination during internal cross-validation, while the Firth model provided directly interpretable adjusted effect estimates with 95% CIs. Model complexity therefore did not confer a clear predictive advantage in this cohort, supporting parsimonious and clinically interpretable approaches for future external validation.

Importantly, the prediction findings should be interpreted within the methodological context of the present study. The sample size of 324 patients remained relatively modest for predictive modeling involving multiple candidate variables and interaction terms. Although clinically guided feature selection, restricted model complexity, five-fold cross-validation, and bootstrap optimism correction were used to reduce overfitting, some optimism may remain. The EIH Firth model had an apparent AUC of 0.854 and an optimism-corrected AUC of 0.845, while pooled out-of-fold AUCs were 0.835 for LASSO, 0.837 for elastic net, and 0.804 for random forest. Thus, penalized logistic approaches matched or exceeded random-forest discrimination, and no single algorithm should be regarded as definitively superior on the basis of this internally validated single-center cohort. These models should therefore be considered exploratory rather than definitive clinical decision-support tools. External validation using larger, multicenter cohorts with diverse patient populations is essential before implementation in routine neurosurgical practice. This cautious interpretation is consistent with current recommendations for the development and reporting of clinical prediction models (26, 27).

Overall, the present findings extend previous work on traumatic hematoma expansion by demonstrating that integration of readily available clinical variables with machine-learning approaches can achieve clinically meaningful prediction of EIH in a resource-limited environment. Unlike many existing prognostic studies that focus primarily on mortality or short-term functional outcomes, our study specifically evaluated hematoma expansion as a dynamic pathological process preceding neurological deterioration. Because EIH represents a potentially modifiable stage in the progression of traumatic intracranial injury, early identification of high-risk patients may facilitate individualized surveillance, prioritization of repeat neuroimaging, optimization of physiological management, and timely neurosurgical intervention. Consequently, the predictive framework developed in this study provides an important foundation for future multicenter validation studies and the eventual development of practical decision-support tools tailored to neurotrauma care in low- and middle-income countries.

Beyond identifying predictors of expansive intracranial hematoma (EIH), this study provides important insights into the spectrum of postoperative outcomes following neurosurgical evacuation in patients with traumatic brain injury (TBI). More than half of the study population experienced at least one postoperative complication, emphasizing the substantial burden of perioperative morbidity associated with severe TBI in this resource-limited setting. Early post-traumatic seizures, cerebral edema, and coma were the most frequent complications, reflecting the complex pathophysiological cascade that follows primary brain injury. These findings are consistent with previous neurotrauma studies demonstrating that secondary neurological complications remain major contributors to prolonged hospitalization, delayed neurological recovery, and increased healthcare utilization despite advances in neurosurgical and critical care management (3, 28, 29). Importantly, the relatively high complication rate observed in our cohort should also be interpreted within the context of delayed presentation, limited prehospital care, constrained intensive care capacity, and restricted access to neurocritical monitoring, factors that continue to challenge trauma systems throughout many low- and middle-income countries (20).

The multivariable analysis identified decompressive craniectomy, delayed surgery performed more than 72 h after injury, and surgery involving frontoparietal, fronto-parieto-temporal, and parieto-temporal regions as independent predictors of intraoperative complications. These associations should not be interpreted as evidence that these procedures or operative locations directly cause complications. Rather, they likely reflect differences in injury severity, lesion complexity, intracranial hypertension, and operative indications that influenced surgical decision-making. Patients undergoing decompressive craniectomy generally presented with extensive cerebral edema, refractory intracranial hypertension, diffuse injury, or impending cerebral herniation, conditions inherently associated with increased operative risk. Likewise, extensive fronto-parieto-temporal injuries frequently involve multiple intracranial compartments, cortical contusions, skull fractures, and major vascular disruption, making surgical management technically more demanding.

The present findings therefore support that operative procedures should be regarded as markers of injury severity rather than independent determinants of adverse outcomes. Similar observations have been reported in the DECRA and RESCUEicp randomized trials, in which decompressive craniectomy effectively reduced intracranial pressure and mortality among selected patients with severe TBI but resulted in a greater proportion of survivors with severe disability, emphasizing the complex balance between survival and long-term neurological recovery (30, 31). Our observational study extends these findings to a sub-Saharan African setting, where treatment decisions must frequently be individualized under considerable resource constraints.

Delayed surgical evacuation beyond 72 h after injury independently predicted intraoperative complications. This finding is biologically plausible because prolonged intervals between injury and definitive intervention permit continued hematoma expansion, progressive cerebral edema, increasing intracranial pressure, and secondary ischemic injury. In Uganda and many other low-resource environments, delayed surgery often reflects multiple system-level barriers, including prolonged transport from peripheral hospitals, delayed diagnosis, limited CT availability, shortages of neurosurgical personnel, and restricted operating theatre capacity (5). Consequently, delayed surgery should not be viewed solely as a patient-related factor but rather as a reflection of broader healthcare system limitations. These findings underscore the importance of strengthening trauma referral networks, improving emergency transport systems, expanding neuroimaging availability, and increasing neurosurgical capacity to facilitate earlier intervention for patients with severe TBI.

Mortality after neurosurgical evacuation remained relatively low compared with many previous reports from sub-Saharan Africa, with an overall mortality of 12.9%. In the parsimonious Firth model, older age, EIH, intraoperative complications, craniotomy, and delayed surgery beyond 72 h were associated with increased mortality. These findings should be interpreted strictly as statistical associations rather than causal relationships. In particular, operative procedure and timing are strongly influenced by clinical presentation, hematoma characteristics, neurological deterioration, physiological instability, referral delay, and surgeon judgement. Residual confounding by indication cannot be excluded despite adjustment for measured covariates, and causal inference is not possible within the observational design of the present study.

A major strength of this investigation is the inclusion of patient-reported quality-of-life outcomes using the Quality of Life after Brain Injury (QoLIBRI) instrument. Although mortality and functional scales remain the most frequently reported endpoints in neurotrauma research, they provide only limited insight into the broader physical, cognitive, emotional, and social consequences experienced by survivors. In the present study, QoLIBRI scores improved progressively throughout follow-up in both patient groups; however, individuals without EIH consistently demonstrated significantly better quality of life at both 90 days and six months after surgery. These findings indicate that hematoma expansion influences not only acute clinical outcomes but also longer-term recovery perceived by patients themselves. Because QoLIBRI specifically evaluates cognition, self-perception, autonomy, emotional well-being, physical health, and social participation, it represents a valuable complement to conventional neurological outcome measures and provides a more comprehensive assessment of recovery following TBI (32, 33).

Multivariable analysis further demonstrated that EIH, intraoperative complications, craniotomy, and delayed surgery were associated with unfavorable quality of life at six months. The very large craniotomy estimate (OR = 65.31, 95% CI 12.67–336.53) should not be interpreted as a causal effect of craniotomy on quality of life. Despite the use of Firth bias-reduced regression to address sparse-data separation, the magnitude and wide confidence interval likely reflect substantial confounding by indication and residual unmeasured severity: patients selected for craniotomy may have had larger or more complex hematomas, greater mass effect, neurological deterioration, or other severity factors not fully captured by the model. Accordingly, craniotomy is better interpreted here as a marker of clinical and injury complexity rather than as a procedure that itself causes poor patient-reported outcome. The associations of EIH and delayed surgery with poorer QoLIBRI outcomes further reinforce the importance of early recognition of progressive hemorrhage, timely reassessment, and appropriate neurosurgical management.

Functional outcome analysis showed that EIH, intraoperative complications, and craniotomy were independently associated with unfavorable functional outcome, whereas decompressive craniectomy showed only a borderline association and delayed surgery was not statistically significant in the adjusted model. These associations should not be interpreted as causal effects of the operative procedures; rather, they likely reflect differences in injury severity, surgical indication, and residual confounding. Overall, the findings support multidimensional assessment of TBI recovery incorporating survival, functional independence, and patient-reported quality of life.

The present study possesses several important strengths. First, the prospective cohort design minimized recall bias and enabled standardized longitudinal follow-up from admission through six months after surgery. Second, serial CT imaging permitted objective identification of EIH using established radiological criteria. Third, the study simultaneously evaluated clinical, physiological, and radiological predictors while integrating conventional multivariable regression with machine-learning approaches, thereby providing complementary perspectives on risk prediction. Fourth, inclusion of both functional and quality-of-life outcomes broadened assessment beyond traditional mortality endpoints. Finally, this investigation represents one of the few prospective studies evaluating predictive modeling for traumatic intracranial hematoma progression in sub-Saharan Africa, thereby contributing valuable evidence from an underrepresented region where the burden of neurotrauma remains disproportionately high.

Several limitations were acknowledged. First, this was a single-center national-referral-hospital cohort, which may limit transportability. Second, although an independent reread of 65 CT examinations demonstrated substantial agreement for EIH classification (*κ* = 0.777) and excellent hematoma-volume reliability (ICC([2,1]) = 0.994), the reliability exercise involved a subset and cannot exclude residual measurement error. Third, the sample size was modest for prediction modeling and only 42 deaths occurred. We therefore collapsed sparse surgical-site categories, reduced the mortality model, used Firth bias-reduced regression, and quantified optimism with bootstrap validation. Fourth, all predictive validation was internal; external multicenter validation is required before clinical implementation. Residual confounding and the observational design also preclude causal interpretation of surgical associations.

The clinical implications of this work are particularly relevant for neurosurgical practice in low-resource settings. The identified predictors of EIH—especially SDH, DAI, blood pressure parameters, and fracture–hematoma interactions—are routinely available during initial assessment and may therefore facilitate practical risk stratification without requiring advanced technologies. The comparable or better internal discrimination of the Firth, elastic-net, and LASSO models relative to random forest supports prioritizing parsimonious and clinically interpretable approaches for future validation. Such models should augment rather than replace clinical judgement and should undergo rigorous prospective external validation before incorporation into routine clinical practice.

Future research should focus on multicenter validation of these predictive models across diverse African and international populations to determine their transportability and calibration. Larger datasets incorporating quantitative hematoma volume measurements, intracranial pressure monitoring, advanced neuroimaging biomarkers, laboratory inflammatory markers, and rehabilitation variables may further improve predictive performance. In addition, future studies should evaluate whether implementation of model-guided surveillance strategies, individualized repeat CT imaging protocols, and earlier neurosurgical intervention can reduce hematoma expansion and improve long-term neurological recovery. The integration of explainable artificial intelligence methods with conventional statistical modeling may also enhance clinical interpretability and facilitate acceptance of machine-learning–based decision-support systems in neurosurgical practice.

## Conclusion

This study demonstrated that expansive intracranial hematoma is a frequent complication following traumatic brain injury in Uganda and remains an important determinant of postoperative recovery and patient-reported quality of life. Readily available clinical and radiological variables successfully predicted hematoma expansion, while internally validated machine-learning models showed promising discriminatory performance for identifying high-risk patients. Although associations between operative procedures and clinical outcomes should not be interpreted causally, the findings highlighted the importance of individualized surgical decision-making, timely neuroimaging, and structured postoperative follow-up. Collectively, these results provided a robust foundation for future multicenter validation studies and support the development of context-specific predictive tools capable of improving neurotrauma care throughout resource-limited settings.

## Data Availability

The raw data supporting the conclusions of this article will be made available by the authors, without undue reservation.

## Supplementary material

The Supplementary Material for this article can be found online at: https://www.frontiersin.org/articles/10.3389/fsurg.2026.1781033/full#supplementary-material

Supplementary Figure 1 Calibration plot for the internally validated EIH Firth model
